# Development of Optical Biosensor Using Protein A-Conjugated Chitosan–Gold Nanoparticles for Diagnosis of Cystic Echinococcosis

**DOI:** 10.3390/bios11050134

**Published:** 2021-04-25

**Authors:** Hanie Safarpour, Hasan Majdi, Ali Masjedi, Abdol Sattar Pagheh, Maria de Lourdes Pereira, Sonia M. Rodrigues Oliveira, Ehsan Ahmadpour

**Affiliations:** 1 Infectious and Tropical Disease Research Center, Tabriz University of Medical Sciences, Tabriz 51666-14766, Iran; hanie.safarpour@yahoo.com; 2 Student Research Committee, Department of Parasitology and Mycology, Tabriz University of Medical Sciences, Tabriz 51666-14766, Iran; 3 Drug Applied Research Center, Tabriz University of Medical Sciences, Tabriz 51666-14766, Iran; hasan90majdi@yahoo.com; 4 Department of Medical Nanotechnology, Faculty of Advanced Medical Sciences, Tabriz University of Medical Sciences, Tabriz 51666-14766, Iran; 5 Immunology Research Center, Tabriz University of Medical Sciences, Tabriz 51666-14766, Iran; ali29masjedi@gmail.com; 6 Department of Immunology, Faculty of Medicine, Tabriz University of Medical Sciences, Tabriz 51666-14766, Iran; 7 Infectious Diseases Research Center, Birjand University of Medical Sciences, Birjand 97178-53577, Iran; satar2011@gmail.com; 8 CICECO-Aveiro Institute of Materials, University of Aveiro, 3810-193 Aveiro, Portugal; sonia.oliveira@ua.pt; 9 Department of Medical Sciences, University of Aveiro, 3810-193 Aveiro, Portugal; 10 HMRI and Hunter Cancer Research Alliance Translational Cancer Research Centres, The University of Newcastle, Callaghan, NSW 2308, Australia

**Keywords:** *Echinococcus granulosus*, hydatidosis, diagnosis, dot-blot, chitosan–gold nanoparticle, protein A, biosensor

## Abstract

Human echinococcosis is a serious parasitic diseasethat still affects millions of people in many parts of the world. Since it can offer a critical threat to people’s health, it is important to discover a rapid, convenient, and economical method for detection. Herein, we propose a novel point of care assay, namely, an enhanced immuno-dot-blot assay for diagnosis of cystic echinococcosis (hydatidosis). This method is based on the formation of a sandwich complex between a goldnanoprobe (chitosan–gold nanoparticleprotein A) and hydatid cyst antigen (Ag B), which holds anti-Ag B antibodies. Briefly, protein A was conjugated to chitosan–gold nanoparticles via glutaraldehyde chemistry. Then, Ag B was immobilized on the surface of a nitrocellulose membrane, which was followed by the addition of the sera sample and gold nanoprobes. The positive signals were easily detectable by naked eye. The signal intensity of this biosensor was proportional to the concentration of active anti-*Echinococcus granulosus* antibodies on the surface of the nanoparticles, titer of antibodies in the sera samples, and concentration of Ag B coated on the nitrocellulose membrane. The minimum concentration to use the protein A for conjugation to detect titer of anti-*Echinococcus* IgGand the concentration of Ag B coated in nitrocellulose membrane were 0.5 and 0.3 mg/mL, respectively. This enhanced immuno-dot-blot assay offers a simple diagnostic technique withoutthe need for expensive equipment for diagnosis of echinococcosis.

## 1. Introduction

Cystic echinococcosis (CE) or hydatid disease is a chronic parasitic infection, which is listed among neglected tropical diseases and caused by the larval stage of *Echinococcus granulosus* (*E. granulosus*) [1]. Transmission of CE to humans occurs by accidental consumption of food or water contaminated with the parasite eggs. After the larvae enter the body, they form one or more cysts in internal organs, mainly in the liver and the lungs and, rarely, in the heart, spleen, kidneys, central nervous system, and bone [2,3]. The disease is widespread globally, with high endemicity in Central Asia, Mediterranean countries, Western China, South America, and Eastern Africa [4,5].

In most cases, the initial stages of infection is asymptomatic; even latent periods of more than 10 years have been reported [6]. Therefore, early detection at a lower cost is still needed for better clinical treatment and disease management. Diagnosis of CE is currently based on three factors: epidemiological history, imaging techniques (ultrasonography, magnetic resonance, and computerized tomography, etc.), and serological methods (indirect fluorescence antibody test (IFAT), enzyme-linked immunosorbent assay (ELISA), indirect hemagglutination (IHA), immunoelectrophoresis, and immunoblotting, etc.) [7,8]. Imaging methods are usually expensive, unavailable in less-developed, remote and secluded areas, and in some cases cannot differentiate between abscess and malignancy with the disease [9]. On the other hand, the conventional serological methods used for human CE diagnosis need specific equipment and expertise and are timeconsuming [10].

Optical biosensors can easily detect with the naked-eye the existence of pathogens in a sample without intricate requirements and analytical devices [11]. Several substances have been used to produce signals in optical biosensors (e.g., optical fiber with an enzyme, antibody, or nucleic acid), but gold nanomaterials are ideal reagents, due to their unique optical attributes [12].

Recently, the conjugation of nanoparticles (NPs) to proteins offers many applications in diagnosis methods [13,14]. Protein A (p.A) is a 42 kDa cell surface protein that shows a high affinity for the Fc-fragment of human IgG and IgGs from various other species [15]. Two different strategies are commonly used for conjugation of proteins to gold nanoparticles (GNPs): classic passive adsorption and covalent conjugation [16,17]. Passive adsorption has several drawbacks, such as desorption of the protein from the gold surface over time and possible impairment of necessary conformational changes required for the antigen-binding site [18]. Alternatively, covalent conjugation of proteins to GNPs minimizes many of the drawbacks. Use of functional polymers is a suitable way for the combination of a protein to GNPs, which along with immobilization and keeping the protein molecules on the surface, can also stabilize the GNPs colloidal suspension [19]. Chitosan (Chi), as a natural polymer polysaccharide (linear polysaccharide), contains hydroxyl and amine groups that can be used for immobilization of biomolecules [20]. Therefore, it has been used in GNPs coating for different biomedical applications. Recent studies have demonstrated the application of chitosan–gold nanoparticles (Chi–GNPs) for biomedical diagnosis. For example, antibody conjugated Chi–GNPs wereengineered for optical detection of Antigen IgG (Ag) in sandwich-like immunoassay systems [21]. In another study, Chi–GNPs were used for colorimetrical detection of *Mycobacterium tuberculosis*’ nucleic acid via physical adsorption to Chi–GNPs [20]. However, up to now, there is no report of p.A conjugated Chi–GNPs for the detection of *E. granulosus*. In the present study, we propose a low-cost optical biosensor as a sensitive tool for the detection of *E. granulosus*.

## 2. Method

### 2.1. Preparation of Hydatid Cysts Fluid

Liver cysts of infected sheep were collected from a local slaughterhouse. The samples were processed in the laboratory and *E. granulosus* fertile cysts were sterilized with alcohol 70%. The crude hydatid cysts fluid (HCF) was aseptically aspirated using a 10 mL syringe. In the next step, the HCF was centrifuged at 10,000× *g* for 30 min for separation of protoscolices (parasite larvae) from the supernatant. Finally, the supernatant was collected, and total protein content was determined via the Bradford assay and confirmed via SDS–PAGE [22]. Samples were then stored at −80 °C until further use.

### 2.2. Preparation of Antigen B

Antigen B (Ag B) was prepared from HCF based on the method previously described by Shirazi et al. (2016) [23]. In this method, 100 mL of HCF was centrifuged at 1500× *g* for 30 min, and the supernatant was dialyzed twice with 0.005 M acetate buffer (pH = 5) for 24 h at 4°C. The dialyzed sample was centrifuged at 30,000× *g* for 30 min at 4 °C; this process allows insoluble proteins (Ag B and Ag 5) to settle. The pellet was dissolved in 10 mL of 0.2 M phosphate buffer, PBS (pH = 8) to eliminate globulins. Then, 2.31 g of ammonium sulfate 40% was added and mixed. After a short timeout, the mixture was centrifuged at 3000× *g* for 30 min. The supernatant was incubated in a water bath for 15 min; in this step, Ag 5 became denatured and insoluble due to its heat sensitivity [24]. Finally, the preparation was centrifuged at 30,000× *g* for 1 h and the supernatant containing Ag B was collected. Then, after filtration (using a 0.2 µm sterile filter) sodium azide, NaN_3_ (x%, x M), was added as a preservative and the mixture was stored at −80 °C. 

### 2.3. Synthesis of Chi–GNPs

**Figure 1:**
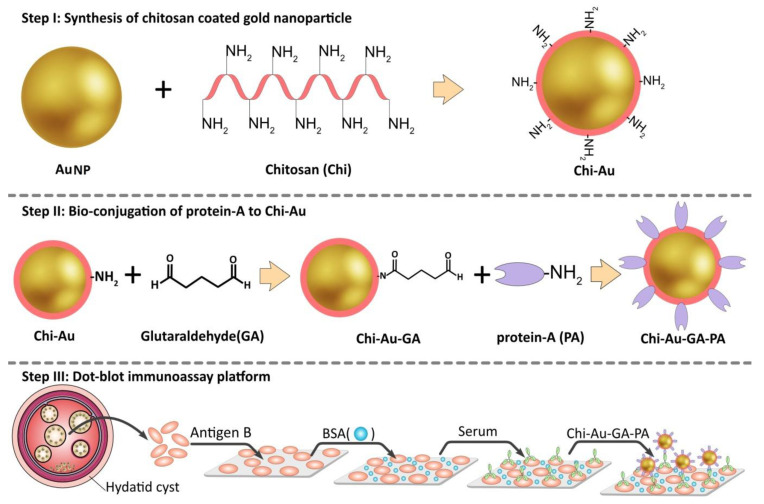
Synthesis of the chitosan–gold nanoparticles followed by complexation with Protein A for sandwich-based *E. granulosus* biosensor. Step I: Colloid gold nanoparticles were synthesized using chitosan. Step II: Chi–GNPs surface was activated by GA and conjugated with proteinA. Step III: Hydatid cyst antigen (Ag B) was immobilized on the NC membrane, membranes were blocked with BSA, and then, treated with serum sample, and finally, each sample was dipped into Chi–GNPs–GA–P.A conjugate.

All required glassware was previously washed with distilled water and sonicated in an ultrasonic bath for 30 min. For Chi–GNPs synthesis (Figure 1. Step I), 500 mg of Chi (molecular weight, 50–190 kDa, DD% 93%, Sigma Aldrich) was dissolved in 50 mL of 1% (*v*/*v*) aqueous solution of acetic acid (Merck), and the reaction was stirred at 65°C by thermostatic magnetic stirring. In the next step, 50 mL of HAuCl_4_ (1 mmol/L tetrachloroauric acid) solution was added to the reaction and kept at 65 °C for 2 h until the color turned wine red. The reaction solution was stirred overnight at 65 °C. The synthesized Chi–GNPs were analyzed using UV–vis spectroscopy. Determination of particle size and zeta potential of the Chi–GNPs were performed by a dynamic light scattering (DLS) instrument (Malvern Zetasizer). Finally, field emission scanning electron microscopy (FE-SEM) studied the Chi–GNPs morphology.

### 2.4. Bio-Conjugation of Protein A on Chi–GNPs Surface

In the present study, we utilized glutaraldehyde (GA) for conjugating p.A with the surface of the Chi–GNPs. The functional aldehyde groups of the GA readily combine with the amine groups of Chi layer on the surface of GNPs. In the first stage, the solution of Chi–GNPs was diluted in distilled water at room temperature, and the pH adjusted to 5.5. Next, 2% GA was added to the nanoparticles’ solution (GA–Chi–GNPs), and left to incubate for 2 h at 40 °C. Then, the mixture was washed three times with 0.01 M PBS (pH 7.4) by centrifugation to eliminate the residual GA. In the next step, 0.3 mL of p.A (1 mg/mL) was combined with0.7 mL of GA–Chi–GNPs solution and incubated for 24 h at 4 °C. Following, the solution was washed again three times with PBS; then, it was incubated with BSA (5%) in PBS (0.01 M, pH 8.5) for 45 min at 20 °C to prevent non-specific binds on the surface of the nanoparticles. In the final step, the conjugate was washed three times with 0.01 M PBS (pH 7.4) to remove physical adsorptions, and then, re-suspended in PBS and stored at 4 °C until further testing (Figure 1, Step II).

### 2.5. Colorimetric Detection

As a control, 0.4 mL p.A-Chi–GNPs and 0.2 mL of PBS (pH = 7.4) were combined and incubated for 20 min at room temperature, then UV–Vis spectra was recorded over the wavelength in the range of 400–800 nm. In the experiment sample, 0.2 mL of human anti-*Echinococcus* IgG solution (1 mg/mL) was added to the 0.4 mL p.A-Chi–GNPs and incubated for 20 min at 25 °C, then UV–Vis absorption spectra was recorded in the wavelength range mentioned in the control.

### 2.6. Dot-Blot Assay

An aliquot of 1μL of each Ag B solution (1.2, 0.6, and 0.3 mg/mL) was spotted on the center of each chamber in nitrocellulose membrane (NC) and allowed to dry for 30 min at 25 °C. Then, membranes were blocked with BSA solution (5% *w*/*v*) and air-dried for 1 h to avoid non-specific binding with the membrane, then washed three times with PBS. In the next step, membranes were incubated with anti-*Echinococcus* IgG (1:1, 1:10 and, 1:100 dilutions) for 1 h at 25 °C and then washed as described before. Finally, membranes were incubated with p.A-Chi–GNPs for 20 min, washed three times with PBS, and dried at room temperature (Figure 1, Step III). At the end, optical results were recorded with a common camera. The numbers of *Toxoplasma gondii* (*T. gondii*) positive sera were used to test for cross-reactivity.

## 3. Results

### 3.1. SDS–PAGE Purified Ag B Characterization

Ag B was successfully isolated, and its total concentration was measured by Bradford assay. The protein concentration of Ag B was 1.2 mg/mL. SDS–PAGE analysis showed bands on 20 and 24 KDa subunits of Ag B (data not shown).

### 3.2. Characterization of the Colloidal Chi–GNPs

**Figure 2:**
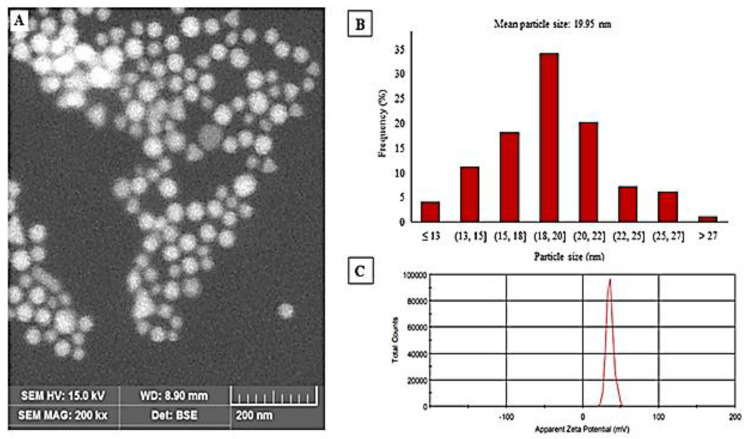
(**A**): SEM image, (**B**): SEM size distribution histogram, and (**C**): Zeta potential of Chi-GNPs.

Colloidal Chi–GNPs solution appears wine red color and the UV–Vis spectrum of Chi–GNPs showed maximum absorption at 522 nm. SEM imaging confirmed that shape and size distribution of Chi–GNPs were slightly homogeneous and non-aggregated with smooth surfaces (Figure 2A). The particle size distribution histogram determined by the SEM images indicated that particles are in the range of 13–27 nm with average diameter size of 19.95 nm (Figure 2B). Chi–GNPs showed an average hydrodynamic diameter of 41.6 nm with a polydispersity index (PDI) of 0.3 and zeta potential of 35.6 mV determined by Zetasizer (Figure 2C).

### 3.3. Conjugation and Characterization of Protein A-Chi-GNPs

We also demonstrate that using of GA for the conjugation of p.A on Chi–GNPs surface resulted in good stability of the Chi–GNPs. In order to find the optimum amount of p.A, different concentrations of 0.25, 0.5, and 1 mg/mL of this protein were used for conjugation on colloidal Chi–GNPs surface. Indeed, by raising p.A concentration, the stability of Chi–GNPs was increased. The UV/Vis spectra of the Chi–GNPs and p.A-Chi–GNPs showed 4 nm shifts in the SPR peak after the conjugation of p.A on Chi–GNPs layer. The conjugation of the p.A on colloidal GNPs can be effectuated a shift from 529 to 532 nm. In addition, HRP protein was used to verify the conjugation of p.A on the Chi–GNPs’ surface. HRP-Tagged-human IgG and HRP-Tagged-human IgM were added to the p.A-Chi–GNPs solution as control. After the addition of substrate (TMB), the blue color appearance of the solution in the presence of HRP-Tagged-human IgG confirmed that the protein- conjugated Chi–GNPs wereactive.

### 3.4. Dot-Blot Immunoassay

**Figure 3:**
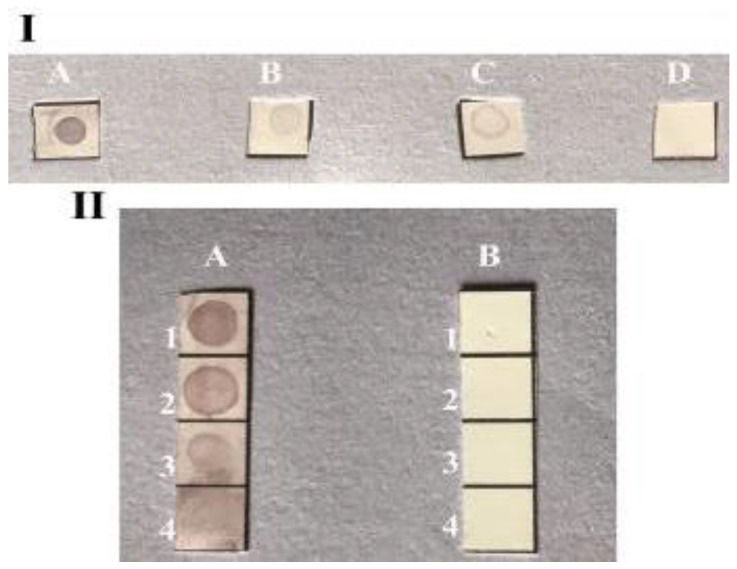
Image of dot-blot immunoassay by 0.5 mg/mL concentration of conjugated p.A on Chi–GNPs surface for detection of antibody at various dilutions. (**I**): (**A**): anti-*Echinococcus* IgG 1:1, (**B**): anti-*Echinococcus* IgG 1:10, (**C**): anti-*Echinococcus* IgG 1:100, (**D**): control. (**II**): (**A**): Ag B, 1:1.2 mg/mL, 2:0.6 mg/mL, 3:0.3 mg/mL, 4:0.15 mg/mL. (**B**): Control.

The principle of this method using dual labeled GNPs (Chi–AuNP-p.A) is based on indirect sandwich of immuno-dot-blot assay (Figure 3). Two concentrations of p.A coupled Chi–GNPs were applied to detect anti-*E. granulosus* antibodies. The color appearance was correlated with the concentration of p.A. The results show that the lowest concentration of anti-*E. granulosus* antibodies can be detected with the 0.5 mg/mL concentration of p.A-coupled Chi–GNPs, in the serum samples. In view of that, this biosensor effectively detected the 1:100 dilution of the anti-*Echinococcus* IgG in 0.3 mg/mL of Ag B. The color intensity decreased with the decline of Ag B concentration. In contrast, there was no color observed in the negative controls. The specificity, efficiency, and the limit of detection were analyzed under the optimum reaction conditions. Three concentrations, 0.3, 0.6, and 1.2 mg/mL, of Ag B spotted in NC membrane and a non-specific antigen *T. gondii* and PBS were used as negative controls. It revealed that the GNPs-based biosensor is able to visually detect a minimal concentration of anti-*Echinococcus* antibodies in real samples.

## 4. Discussion

Echinococcosis is a neglected serious parasitic disease that affects millions of individuals worldwide and manifests devastating effects on animal husbandry. Recent studies highlighted on various aspects of *E. granulosus* such as world distribution, pathogenesis, diagnostic methods, and novel therapeutic in humans and animals [1,25]. Although there are various methods for diagnosis of this zoonosis parasite, many of these tests requirespecialist training and strict infrastructure demands, with a relativelyhighcost. Among the common methods, the imagining techniques are used to screen the population at a relatively inexpensive cost, such asultrasonography and radiology (X-ray) [1]. Furthermore, the serology tests are also widely used for detection of markers from the parasite (circulating antigens or DNA) and from the host (markers of inflammation, cytokines, or chemokines) [9,26]. Today, clinicians can follow specific guides and algorithms for the diagnosis and treatment of echinococcosis [1,27]. The laboratory diagnosis of cystic echinococcosis (CE) includes, among others, the detection of antibodies, antigens, and cytokines. However, these approaches are not ideal as valid diagnostic tools, given the lack of sensitivity and/or sufficient specificity [28]. Moreover, they require specific infrastructure settings and trained personnel.

Currently, with the advent of nanotechnology, a great advance has been possible in the development of biosensors for the diagnosis of echinococcosis. Li et al. (2017) [29] reported on an optical biosensor for the detection of cystic hydatid disease based on the near-infrared transmission angular spectra of porous silicon microcavity, as a useful method. More recently, in silico design and evaluation was reported as a suitable method of hydatid cyst antigens diagnosis [30]. Notably, researchers have been developing portable electroanalytical biosensing analyzers or devices, in order to efficiently, effectively, and quickly detect pathogens. Despite substantial progress in recent years, more simple and fast techniques are still needed urgently. Thus, nanoparticle-based biosensors are highlydesirablefor their chemical and mechanical properties, which have capabilities in human and veterinary medicine [31,32].

The present study proposes a simple, fast, and advantageous method for the diagnosis of echinococcosis. This method is based on the formation of a sandwich complex between a goldnanoprobe (chitosan–gold nanoparticleprotein A) and hydatid cyst antigen (Ag B), which holds anti-Ag B antibodies. The colorimetric results are achieved fast by visual a change in colors, which remain unchanged upon the negative presence of Ag B antibodies. It is noteworthy that we present a field-applicable method based on a blood sample for rapid diagnosis of infected cases, without needingto expert individuals and advanced equipment.

It is also well demonstrated that gold nanoprobes have been widely used for biosensing, particularly via DNA detection. In an elegant study, a multiplex non-cross-linking colorimetric methodology approach was used for detection of malaria and tuberculosis pathogens [33]. Our research in this direction has given fruitful results, by using chitosan–gold nanoparticles protein A and Ag B. This novel colorimetric biosensor approach was demonstrated to quickly and effectively detect *E. granulosus* infection. Chitosan has been previously used to optimize the synthesis of gold nanoparticles and to develop distinct color changes [34]. Chitosan-capped gold nanoparticles or gold nanoparticles functionalized or stabilized with organic polymers such as chitosan nanocomposites have been described as optimal delivery systems, avoiding the toxicity inherent to other chemicals reagents [35,36].

To summarize, we described here for the first time a fast, stable, and cost-effective biosensor with a colorimetric reading for detecting *E. granulosus* infections. In fact, chitosan-based biosensors have reported good selectivity, sensitivity, and stability for the detection of various targets including glucose, proteins, DNAs, bacteria, and several small biomolecules [37,38]. Remarkably, colorimetric biosensors and gold nanoparticle colorimetric biosensors have shown significant applications in diagnostics [39,40]. Here, we gathered the current knowledge in nanotechnology and colorimetric biosensors to develop this novel Echinococcosis biosensor that can help clinicians and professionals in the field for screening populations and quickly address potential epidemic focus.

## 5. Conclusions

As a conclusion, the GNPs (or AuNPs)-based biosensor was designed for visual detection of minimal concentration of anti-*Echinococcus* IgG in human and animal blood samples for the diagnosis of cystic echinococcosis. In the future, simple and affordable biosensors will be in high demand, as their possible in situ applications will be very important for screening and controlling epidemic and pandemic situations. These portable devices will be particularly valuable where strictly controlled infrastructures are not accessible or possible. The proof-of-concept here described and characterized may easily translate into practical settings that allow fast and simple Au-nanoprobe-based detection on-demand.

## Data Availability

Not applicable.
